# Impact of submandibular gland preservation in neck management of early-stage buccal squamous cell carcinoma on locoregional control and disease-specific survival

**DOI:** 10.1186/s12885-020-07534-5

**Published:** 2020-10-27

**Authors:** Bo Gu, Qigen Fang, Yao Wu, Wei Du, Xu Zhang, Defeng Chen

**Affiliations:** 1 Department of Ultrasound, Affiliated Cancer Hospital of Zhengzhou University, Henan Cancer Hospital, Zhengzhou, Henan People’s Republic of China; 2 Department of Head Neck and Thyroid, Affiliated Cancer Hospital of Zhengzhou University, Henan Cancer Hospital, Zhengzhou, Henan People’s Republic of China

**Keywords:** Submandibular gland preservation, Oral squamous cell carcinoma, Buccal squamous cell carcinoma, Early-stage tumor, Survival analysis

## Abstract

**Background:**

The feasibility of submandibular gland (SMG) preservation in oral squamous cell carcinoma (SCC) has occasionally been analyzed, but the differences in survival associated with the presence or absence of SMG preservation remain unknown. We aimed to prospectively evaluate the oncologic results of SMG preservation in cT1-2 N0 buccal SCC.

**Methods:**

This was a prospective, non-randomized cohort study. Patients with surgically treated cT1-2 N0 buccal SCC were prospectively enrolled and divided into two groups based on the management of the SMG. Level 1b lymph nodes were categorized into six groups based on the positional relationship between the lymph node and the SMG. The main study endpoints were locoregional control (LRC) and disease-specific survival (DSS).

**Results:**

A total of 31 of the 137 included patients underwent SMG-sparing neck dissection. Patients with SMG preservation were likely to be young persons. Superior metastasis occurred in 11 patients with a prevalence of 8.0%, followed by an anterior metastasis rate of 5.1%, and no metastases developed deeply or within the SMG. The 5-year LRC rates in the SMG-sparing and SMG-excision groups were 74 and 75%, respectively, and the difference was not significant (*p* = 0.970). The 5-year DSS rates in the SMG-sparing and SMG-excision groups were 74 and 69%, respectively, and the difference was not significant (*p* = 0.709).

**Conclusions:**

SMG involvement was rare, and the superior group carried the highest risk for lymph node metastasis. SMG-sparing neck dissection is selectively suggested in cT1-2 N0 buccal SCC patients, and could avoid postoperative asymmetric appearance and dry mouth.

**Supplementary information:**

**Supplementary information** accompanies this paper at 10.1186/s12885-020-07534-5.

## Background

Buccal squamous cell carcinoma (SCC) is the second most common malignancy of the oral cavity in China [1]; surgical excision is the main treatment, and neck dissection is an important part of surgery even for early-stage (cT1-2 N0) disease, owing to the lack of an anatomic barrier and the relatively high cervical metastatic rate [2–4].

Since its first description in lymphadenectomy by Crile et al. [5] in 1906, submandibular gland (SMG) excision is usually a standard process to clear all possible lymph nodes in level 1b in any type of neck dissection for oral SCC based on the fact that the SMG is located adjacent to the primary tumor and that there are possible lymph nodes within the SMG. With the progression of anatomy research and surgical techniques, many authors have confirmed that there are no lymph nodes within the SMG [6–8]. Considering the important function of the SMG, which is responsible for 70% of unstimulated saliva production, current evidence has explored the feasibility of SMG preservation in cT1-2 N0 oral SCC [9–21]. Most researchers support that SMG-sparing neck dissection is safe under the condition that the primary tumor is small and not close to the SMG by retrospectively analyzing the metastatic pattern in level 1b. Unfortunately, very few authors have demonstrated a survival difference associated with the presence or absence of SMG preservation [22, 23], which represents the most important evidence.

Usually, the buccal anatomic site is far from the SMG; therefore, in our study, we aimed to prospectively analyze the oncologic safety of SMG preservation in cT1-2 N0 buccal SCC.

## Methods

### Ethnic consideration

The Zhengzhou University institutional research committee approved our study, and all participants signed an informed consent agreement. All procedures performed in studies involving human participants were in accordance with the ethical standards of the institutional and/or national research committee and with the 1964 Helsinki declaration and its later amendments or comparable ethical standards.

### Patient selection

**Fig. 1:**
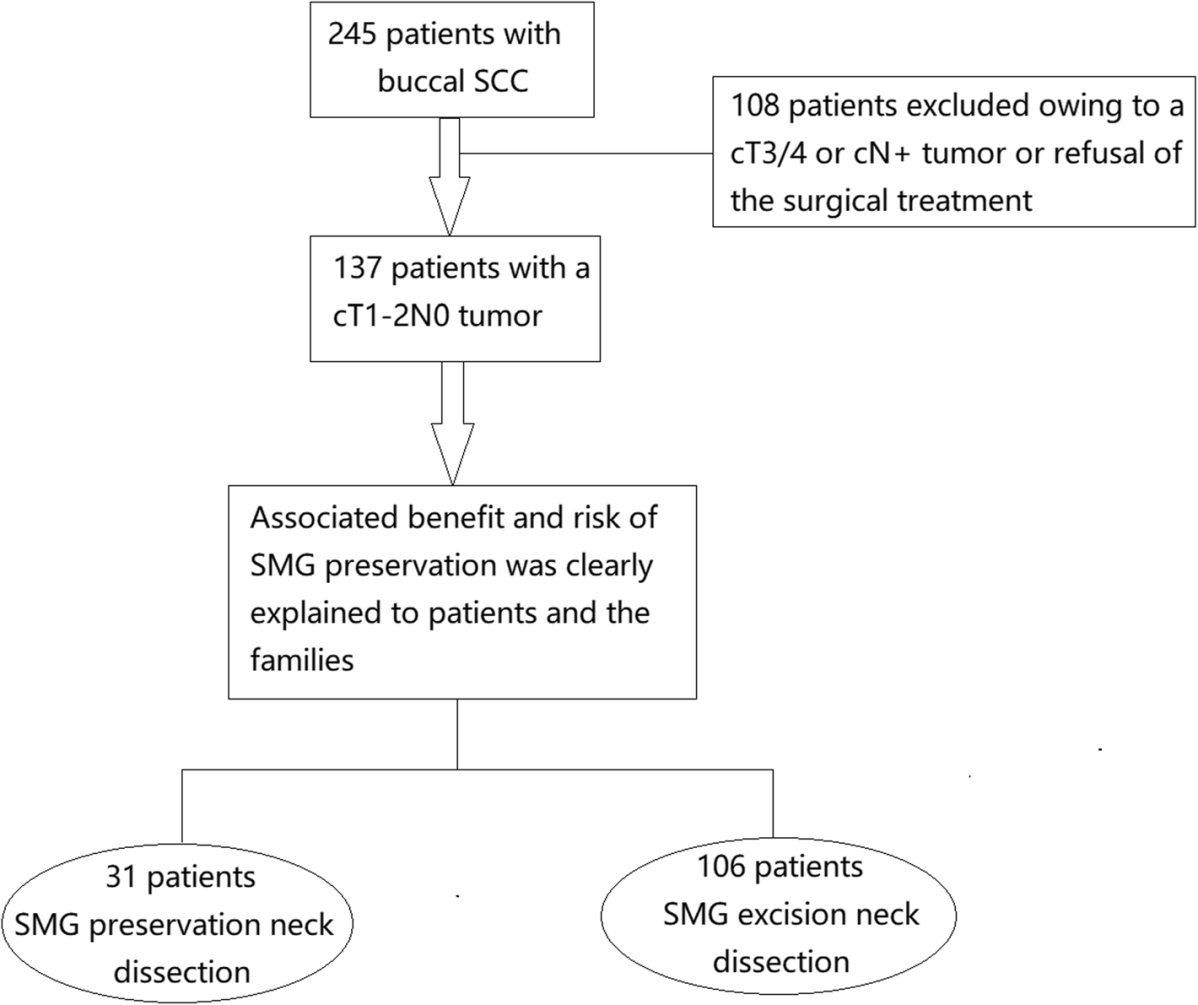
A flow chart for the prospective selection of patients

From January 2012 to December 2018, a total of 245 consecutive patients with buccal SCC sought medical services in our department, and 137 patients were diagnosed with a cT1-2 N0 tumor after systemic examinations. There are no official guidelines for SMG management during neck dissection in China. In our cancer center, we have tried to preserve the SMG during neck dissection starting in 2012 in selected patients. The decision regarding SMG management was based on preoperative communication with the patient and his/her family members, and the associated benefits including avoidance of asymmetric appearance and decreased possibility of dry mouth and risks were clearly explained to them. Thirty-one patients decided to have SMG preservation during neck dissection, and 106 patients decided to have a traditional neck dissection (Fig. 1). The 137 patients’ data regarding age, sex, TNM stage based on the 8th AJCC system, pathologic characteristics, and follow-up were collected and analyzed.

### Definition of important variables

All patients underwent preoperative systemic ultrasound and CT/MRI examinations, and a cN0 neck was defined as the absence of clinically suspicious lymph nodes based on palpation and image analysis. A cT1/cT2 tumor was defined as a tumor with a maximum diameter less than 2 cm or ranging from 2 cm to 4 cm with a depth of invasion (DOI) less than 10 mm in MRI. Drinkers were defined as those who consumed at least one alcoholic drink per day for at least 1 year, and smokers were defined as those who smoked on a daily basis or who had quit smoking for less than 5 years [24]. Perineural invasion (PNI) was considered to be present if tumor cells were identified within the perineural space and/or nerve bundle; lymphovascular infiltration (LVI) was positive if tumor cells were noted within the lymphovascular channels [25]. The pathologic DOI was measured from the level of the adjacent normal mucosa to the deepest point of tumor infiltration, regardless of the presence or absence of ulceration [26].

### Treatment proposal

**Fig. 2:**
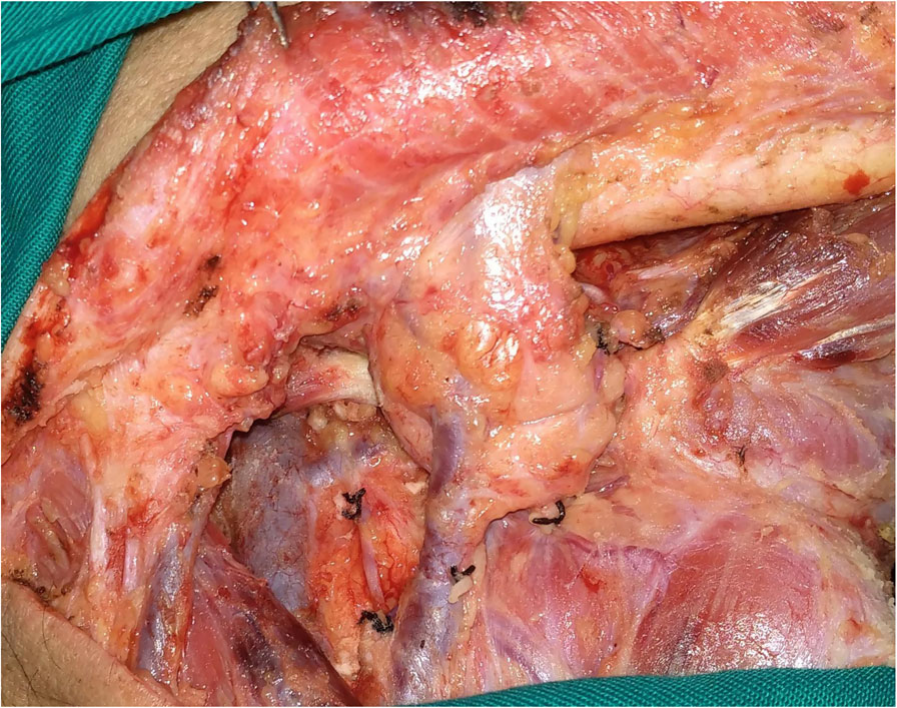
Neck dissection with submandibular gland preservation in a cT2 buccal squamous cell carcinoma patient

**Fig. 3:**
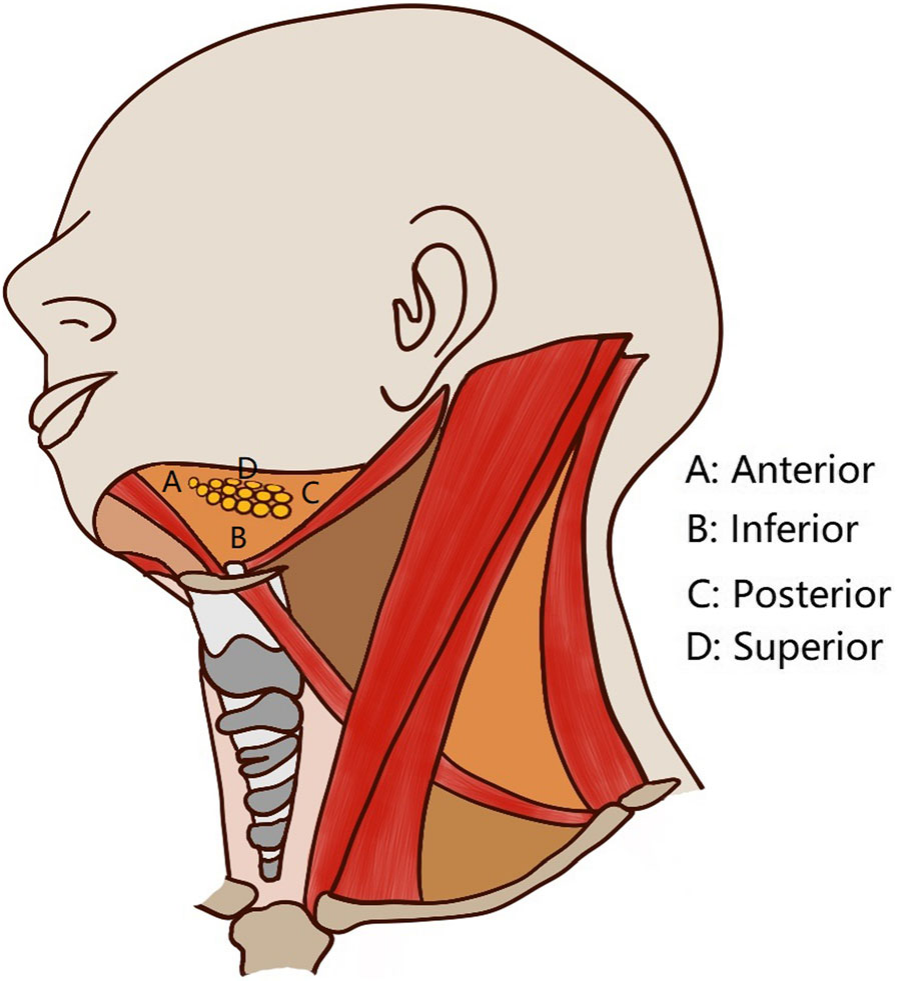
Diagram of the six groups of level 1b lymph nodes

In all patients, neck dissection was performed first via a submandibular incision approach, and lymph nodes from levels 1 to 3 were dissected. For SMG-sparing neck dissection (Fig. 2), lymphoid and adipogenic tissues around the SMG in the level 1b except the tissues located deeply were resected while maintain the integrity of the SMG capsule. The level 1b specimens were divided into the following 5 groups based on their positional relationship with the SMG and were then sent for separate pathologic analysis (Supplemental Fig. 1 and Supplemental Fig. 2): anterior: the tissue located anterior to the SMG; posterior: the tissue located posterior to the SMG; superficial: the tissue located superficial to the SMG; superior: the tissue located superior to the SMG; and inferior: the tissue located inferior to the SMG (Fig. 3). For neck dissection with SMG excision, the level 1 specimens were similarly divided into 6 groups, and these tissues as well as the SMG were then sent for separate pathologic analysis. The newly added sixth group (deep) referred to the tissue located deep to the SMG. The primary tumor was completely resected with a margin of at least 1 cm by a transoral route. Adjuvant treatment was suggested if there was a positive margin, pathologic neck node metastasis, PNI, or LVI. After discharge, the patient was followed every 3 months for the first two years, every 6 months for the third to fifth years after the operation, and then once per year thereafter. Active interference was performed if disease recurrence was suspected.

### Statistical analysis

The Chi-square test was used to compare the categorized variables between the two groups, and Student’s t test was used to compare the continuous variables between the two groups. The study endpoints were locoregional control (LRC) and disease-specific survival (DSS). The Kaplan-Meier method was used to calculate the survival rates, and the LRC survival time was calculated from the date of surgery to the date of first disease recurrence or the last follow-up. The survival time of DSS was calculated from the date of surgery to the date of cancer-related death or the last follow-up. A Cox proportional hazards model was used to determine the independent prognostic factors. All reported *p* values were two-sided, and *p* < 0.05 was considered to be significant. All statistical analyses were performed using SPSS 20.0.

## Results

### Demographic information

A total of 137 patients were enrolled for analysis. In 31 (22.6%) patients undergoing SMG-sparing neck dissection, there were 20 (64.5%) males and 11 (35.5%) females, and the mean age was 55.4 years with a range from 38 to 65 years. Smokers and drinkers were noted in 19 (61.3%) and 15 (48.4%) patients, respectively. Transient marginal mandibular nerve injury occurred in 2 (6.5%) patients. In 106 (77.4%) patients with SMG excision, there were 83 (78.3%) males and 23 (21.7%) females, and the mean age was 60.5 years with a range from 35 to 78 years. Smokers and drinkers were noted in 79 (74.5%) and 57 (53.8%) patients, respectively. Transient marginal mandibular nerve injury occurred in 10 (9.4%) patients. The two groups had significant differences in age (*p* = 0.034), no significant difference regarding other variables was noted (all *p* > 0.05) (Table 1).

**Table 1:** Comparison of demographic and pathologic variables between the submandibular gland (SMG) preservation and SMG excision groups

Variables	SMG preservation	SMG excision	*p*
	*N* = 31	*N* = 106	
Age	55.4 (38–65)	60.5 (35–78)	0.034
Sex			
Male	20 (64.5%)	83 (78.3%)	
Female	11 (35.5%)	23 (21.7%)	0.118
Smoker			
Yes	19 (61.3%)	79 (74.5%)	
No	12 (38.7%)	27 (25.5%)	0.151
Drinker			
Yes	15 (48.4%)	57 (53.8%)	
No	16 (51.6%)	49 (46.2%)	0.597
Clinical tumor stage			
T1	17 (54.8%)	45 (42.5%)	
T2	14 (45.2%)	61 (57.5%)	0.223
Pathologic tumor stage			
T1	15 (48.4%)	40 (37.7%)	
T2	16 (51.6%)	66 (62.3%)	0.287
Perineural invasion			
Positive	4 (12.9%)	10 (9.4%)	
Negative	27 (87.1%)	96 (90.6%)	0.736
Lymphovascular invasion			
Positive	3 (9.7%)	10 (9.4%)	
Negative	28 (90.3%)	96 (90.6%)	1.000
Tumor differentiation			
Well	10 (32.3%)	30 (28.3%)	
Moderate	14 (45.2%)	64 (60.4%)	
Poor	7 (22.6%)	12 (11.3%)	0.199
Cervical lymph node stage			
N0	25 (80.6%)	88 (83.0%)	
N+	6 (19.4%)	18 (17.0%)	0.760
Transient MMN injury*	2 (6.5%)	10 (9.4%)	0.734
Postoperative radiotherapy	4 (12.9%)	23 (21.7%)	0.319

^*^MMN Marginal mandibular nerve

### Pathologic variables

In the SMG-sparing group, cT1 and cT2 tumors were characterized in 17 (54.8%) and 14 (45.2%) patients, respectively. Two cT1 tumors were corrected as pT2 tumors after the operation. The median pathologic DOI was 5.1 mm with a range from 1.0 to 9.5 mm. Negative margins were achieved in all patients. PNI and LVI were noted in 4 (12.9%) and 3 (9.7%) patients, respectively. Tumor differentiation was good in 10 (32.3%) patients, moderate in 14 (45.2%) patients, and poor in 7 (22.6%) patients. Cervical node metastasis occurred in 6 (19.4%) patients (Table 1). There was no extracapsular spread; the total number of positive lymph nodes was 7, 1 patient had one level 1a pathologic node and one level 1b node, and 5 patients had isolated level 1b pathologic lymph nodes. According to further subgroup analysis, superior metastasis occurred in 3 patients, anterior metastasis occurred in 2 patients, and posterior metastasis occurred in 1 patient (Table 2).

**Table 2:** Metastasis pattern in level 1b in patients with submandibular gland (SMG) preservation and SMG excision

Groups of level 1b	SMG preservation (*n* = 6)	SMG excision (*n* = 18)	Overall rate (/137)
Superior	3	8	8.0%
Anterior	2	5	5.1%
Posterior	1	3	2.9%
Inferior	0	1	0.7%
Superficial	0	1	0.7%
Deep	–	0	0.0%
SMG	–	0	0.0%

In the SMG-excision group, cT1 and cT2 tumors were characterized in 45 (42.5%) and 61 (57.5%) patients, respectively. Five cT1 tumors were corrected as pT2 tumors after the operation. The median pathologic DOI was 5.6 mm with a range from 1.5 to 9.8 mm. Negative margins were achieved in all patients. PNI and LVI were noted in 10 (9.4%) and 10 (9.4%) patients, respectively. Tumor differentiation was good in 30 (28.3%) patients, moderate in 64 (60.4%) patients, and poor in 12 (11.3%) patients. Cervical node metastasis occurred in 18 (17.0%) patients (Table 1). There was no extracapsular spread; the total number of positive lymph nodes was 21: 15 patients with N1 stage lymph nodes and 3 patients with N2 stage lymph nodes. According to further subgroup analysis, superior metastasis occurred in 8 patients, anterior metastasis occurred in 5 patients, posterior metastasis occurred in 3 patients, superficial metastasis occurred in 1 patient, and inferior metastasis occurred in 1 patient. No metastases developed deeply or within the SMG (Table 2).

The overall metastasis rates in the superior and anterior groups were 8.0 and 5.1%, respectively. The two groups had similar distributions regarding clinical tumor stage (*p* = 0.223), pathologic tumor stage (*p* = 0.287), PNI (*p* = 0.736), LVI (*p* = 1.000), and pathologic neck metastasis (*p* = 0.760) (Table 1).

### Survival data

During our follow-up with a mean time of 40.5 (range: 7–88) months, in the SMG-sparing group, 4 patients received adjuvant radiotherapy, 1 patient had local recurrence, and 6 patients had regional recurrence: level 1 occurrence in 2 patients, level 2 in 3 patients, and level 3 in 3 patients. There were no cases of distant metastasis; 3 patients with disease recurrence underwent successful salvage treatment by radical surgery, and 5 patients died of the disease.

In the SMG-excision group, 23 patients received adjuvant radiotherapy, 4 patients had local recurrence, and 19 patients had regional recurrence: level 1 occurrence in 5 patients, level 2 in 8 patients, level 3 in 8 patients, and level 4 in 2 patients. There were no cases of distant metastasis, 10 patients with recurrent disease underwent successful salvage treatment by radical surgery, and 19 patients died of the disease.

**Fig. 4:**
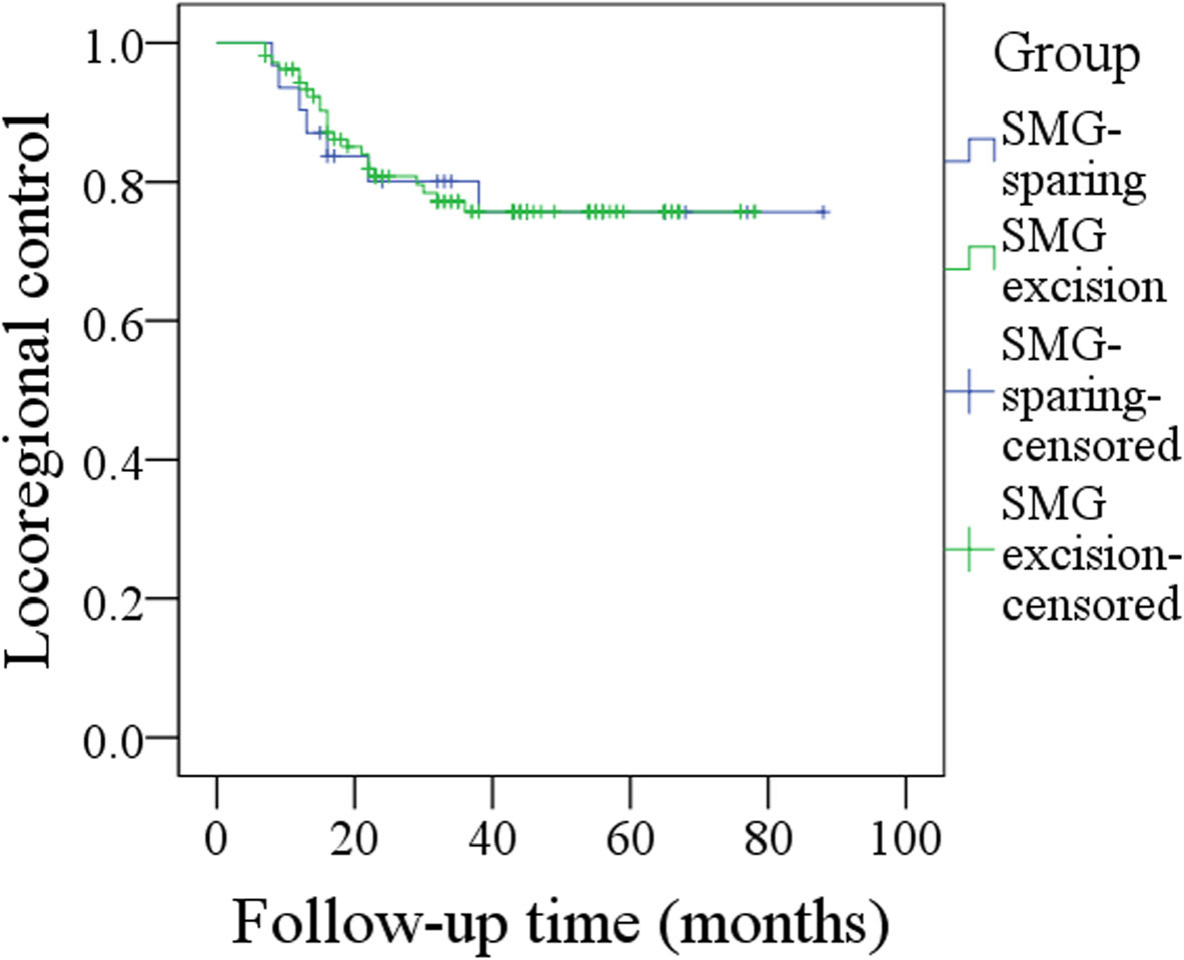
Comparison of locoregional control survival in patients with or without submandibular gland excision (*p* = 0.970)

**Fig. 5:**
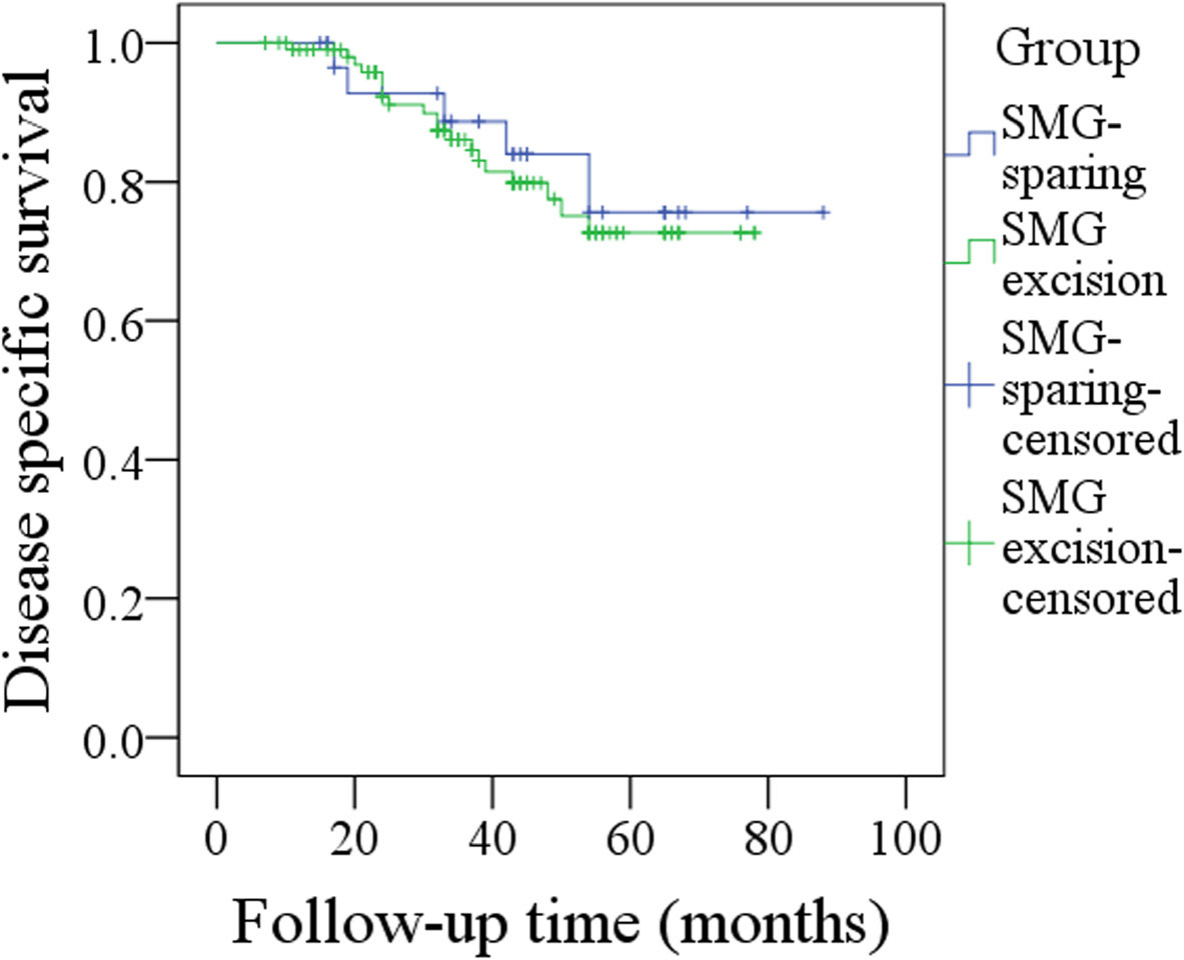
Comparison of disease-specific survival in patients with or without submandibular gland excision (*p* = 0.709)

The 5-year LRC rates of the SMG-sparing and SMG-excision groups were 74 and 75%, respectively, and the difference was not significant (*p* = 0.970, Fig. 4). The 5-year DSS rates of the SMG-sparing and SMG-excision groups were 74 and 69%, respectively, and the difference was not significant (*p* = 0.709, Fig. 5).

## Discussion

The most important finding in the current study was that there was no difference in survival regarding the presence or absence of SMG preservation. To the best of our knowledge, this is the first prospective evidence that supports the reliability of SMG preservation in cT1-2 N0 buccal SCC; these findings alleviate the concerns regarding the possible decreased disease control caused by SMG preservation and improve patients’ postoperative quality of life including avoidance of asymmetric appearance and decreased possibility of dry mouth.

Both level 1b lymph nodes and the SMG are located in the submandibular region, and level 1b lymph nodes are usually the first and also the most common metastatic site in oral SCC. The assessment of SMG-involved patterns in level 1b lymph nodes may provide the theoretical basis for SMG preservation. In a study consisting of 236 patients with oral SCC by Basaran et al. [9], 13 (4%) patients had SMG involvement, and the most common pattern was direct invasion by the primary tumor, which occurred in 8 patients with a T3–4 tumor of the tongue or the floor of the mouth. Invasion by metastatic lymph nodes developed in 4 patients, of whom 2 had a T2 tumor, and metastatic disease was confirmed within the SMG in 1 patient. The finding of metastatic disease within the SMG was interesting, but the authors did not give a detailed description of this specific case; thus, it remains unknown whether there was a suspicious lesion according to preoperative examinations. Byeon et al. [10] reported that just 2 (1.0%) of 201 patients with oral SCC showed SMG involvement, and the only pattern was direct extension from a primary tumor of the floor of the mouth and the retromolar trigone. Cakir Cetin et al. [11] also reported that only 2 (1.3%) patients had SMG involvement, and both of them had invasion by a T4 SCC tumor in the tongue or the floor of the mouth. A recent review by Dundar et al. [21] described that 58 resected SMGs of 2792 cases revealed to have tumor involvement, and direct SMG invasion by the primary tumor accounted for nearly 75% of the common invasion pathway. Similar results were also reported by Chen et al. [12] and Subramaniam et al. [19]; moreover, these authors noted that the characteristics of a primary site of the tongue or floor of the mouth, the presence of T3/T4 disease, and an advanced neck nodal status were related to a significantly increased probability of SMG involvement. These findings may well explain our finding: there was no direct or secondary SMG involvement in the current study.

The surgical technique required for SMG preservation is not a concerning matter [13], and our head and neck department performs nearly 5000 operations every year; thus, our rich experience enables us to face many difficulties [24, 25, 27–30]. The biggest problem involves clearing any possible lymph nodes around the SMG. Rouviere et al. [31] divided level 1b lymph nodes into five groups: preglandular lymph nodes, prevascular lymph nodes, retrovascular lymph nodes, retroglandular lymph nodes, and intracapsular submandibular lymph nodes. Afterwards, a sixth group of deep submandibular lymph nodes was described by DiNardo et al. [8]. A few authors have analyzed the metastasis rate of these subgroups with some modifications. In a report by Lim et al. [32], 41 patients with cN0 tongue SCC underwent 72 neck dissections, and positive perivascular lymph nodes occurred in four (5.6%) of the 72 procedures; 14 patients with cN0 SCC of the floor of the mouth underwent 27 neck dissections, and positive perivascular lymph nodes occurred in two (7.4%) of the 27 procedures. In another study published by Agarwal et al. [33], the authors found that in 231 patients with cN0 oral SCC, the incidence of positive perivascular lymph nodes was 7.1% in tongue SCC and 7.8% in buccal SCC, and the difference was not significant. The perivascular lymph nodes in the abovementioned two studies referred to the prevascular lymph nodes and the retrovascular lymph nodes and were similar to the superior group of lymph nodes in the current study. Our rate of metastasis in the superior group was consistent with previous findings, and it was noted that this group carried the highest risk for metastatic lymph nodes. Meticulous manipulation was required for this area when performing SMG-sparing neck dissection. Literature regarding metastasis in other groups is extremely rare. Malik et al. [34] might be the only researcher to analyze the submandibular subgroup metastasis pattern in patients with oral SCC; in their 32 patients with cN0 buccal SCC, 3 (9.4%) patients had superior metastasis, and 2 (6.5%) patients had anterior metastasis, and there were no metastases in the other subgroups. Similar findings were also noted in the current study.

Deep submandibular lymph nodes and intracapsular submandibular lymph nodes are the most important lymph nodes, and both groups attract the most concern. These two groups of lymph nodes are usually left behind during SMG-sparing neck dissection. Deep submandibular lymph nodes were introduced in an anatomic study by DiNardo et al. [8]; these lymph nodes were small and inconsistently present and were located anywhere posterior to the hyoglossus muscle or superficial to the mylohyoid muscle but along the undersurface of the submandibular gland. Unfortunately, the authors failed to present the incidence of these lymph nodes. In studies by Dhiwakar et al. [13] and Yang et al. [6] as well as in our study, there were no deep lymph nodes. This finding suggests that deep submandibular lymph nodes might exist, but their incidence is extremely low.

There remains great controversy regarding intracapsular submandibular lymph nodes. Although some researchers have reported the occurrence of intracapsular submandibular lymph node metastasis in oral SCC [12, 34], its prevalence is very low, less than 0.7%, and it is mostly caused by advanced-stage disease. Currently, increasing evidence, including our findings, has shown that the SMG does not contain a rich network of lymphovascular structures and lymph nodes [10, 17]. Metastasis in the SMG is generally regarded to occur from primary tumors located outside the head and neck region via the hematogenous route [14, 18].

Survival analysis provides the strongest evidence for SMG preservation. However, only two studies were available for comparison. Chen et al. [22] might have been the first to analyze the impact of SMG preservation on survival. There were 408 patients with cT1-2 N0 oral SCC included, and 33 patients had SMG preservation during neck dissection. Of the 33 patients, 8 were diagnosed with buccal SCC. The authors reported that among patients with buccal SCC, the 5-year disease-free survival rates of those with and without SMG preservation were 75 and 69%, respectively, and the difference was not significant (*p* = 0.83); the 5-year overall survival rates of these two groups were 87.5 and 95.6%, respectively, and the difference was also not significant (*p* = 0.54). However, as the study stated itself, the number of patients undergoing SMG-sparing neck dissection was very small, and some possible adverse pathologic factors were under-detected. In a paper by Lanzer et al. [23], the authors enrolled 66 patients who had SCC of the floor of the mouth or the tongue; 21 patients underwent SMG-sparing neck dissection, and 28.6% of these patients developed regional recurrence, which was significantly influenced by SMG excision. Based on this finding, the authors concluded that the SMG must be excised for SCC of the floor of the mouth and tongue. However, this study consisted of patients with stage 1–4 tumors. For stage 3 and 4 tumors, postoperative radiotherapy was required; SMG preservation in such cases was meaningless and even decreased disease control. Usually, most studies suggest that it is wise to preserve the SMG in tumors that are likely to be node-negative and do not require adjuvant radiotherapy to achieve both oncologic safety and functional results [14–16]. In the current study, we were the first to describe patients with SMG preservation who had similar LRC and DSS to patients without SMG preservation. This finding was significant and provided the most powerful evidence for SMG preservation so far [35].

Limitations in the current study must be acknowledged: first, although this was a prospective study, it lacked randomization, and there was unavoidable or unnoticed selection bias; second, the two groups had some differences regarding the demographic variables, which might be explained by the fact that young patients might care more about postoperative appearance and xerostomia, but it might introduce interference, and more high-quality studies are needed; thirdly, our sample size was relatively small, then its statistic power was limited, further research was required.

## Conclusions

In summary, SMG involvement was rare, and the superior group carried the highest risk for lymph node metastasis. SMG-sparing neck dissection is selectively suggested in cT1-2 N0 buccal SCC patients.

## Supplementary information

**Additional file 1 Supplemental Figure 1**. The anterior and superior parts were resected.

**Additional file 2 Supplemental Figure 2**. The Posterior and superior parts were resected.

## Data Availability

All data generated or analyzed during this study are included in this published article. And the primary data could be achieved from the corresponding author.

## Abbreviations

SCC: Squamous cell carcinoma
SMG: Submandibular gland
LRC: Locoregional control survival
DSS: Disease specific survival
DOI: Depth of invasion
PNI: Perineural invasion
LVI: Lymphovascular infiltration
