# Supplementary figures and images for: Impact of submandibular gland preservation in neck management of early-stage buccal squamous cell carcinoma on locoregional control and disease-specific survival
Source: BMC Cancer. 2020 Oct 27;20:1034. doi: 10.1186/s12885-020-07534-5 (PMC7592590; doi:10.1186/s12885-020-07534-5)

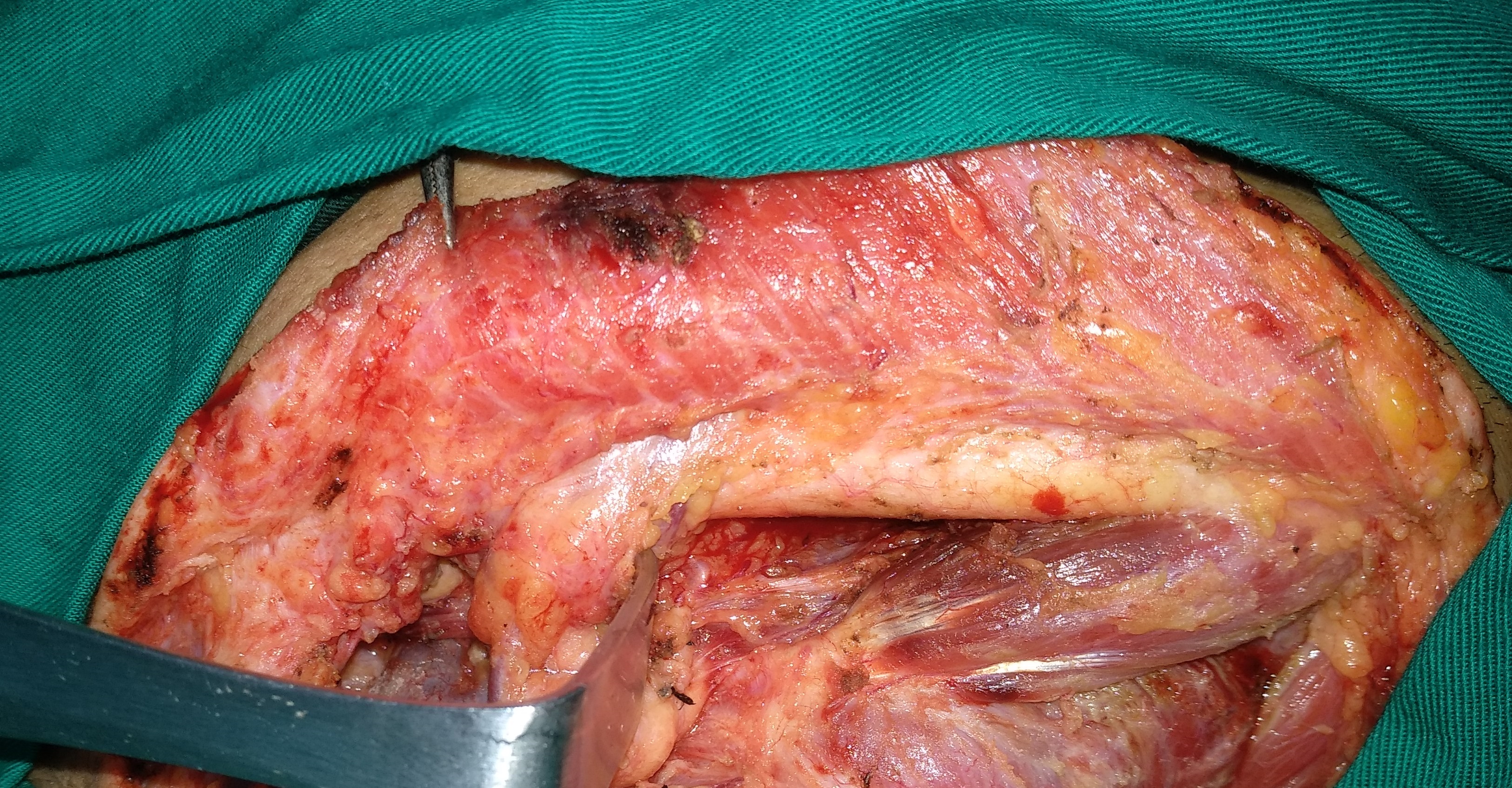

Supplement: Supplementary file 1 — Additional file 1 Supplemental Figure 1. The anterior and superior parts were resected. [file 12885_2020_7534_MOESM1_ESM.jpg]

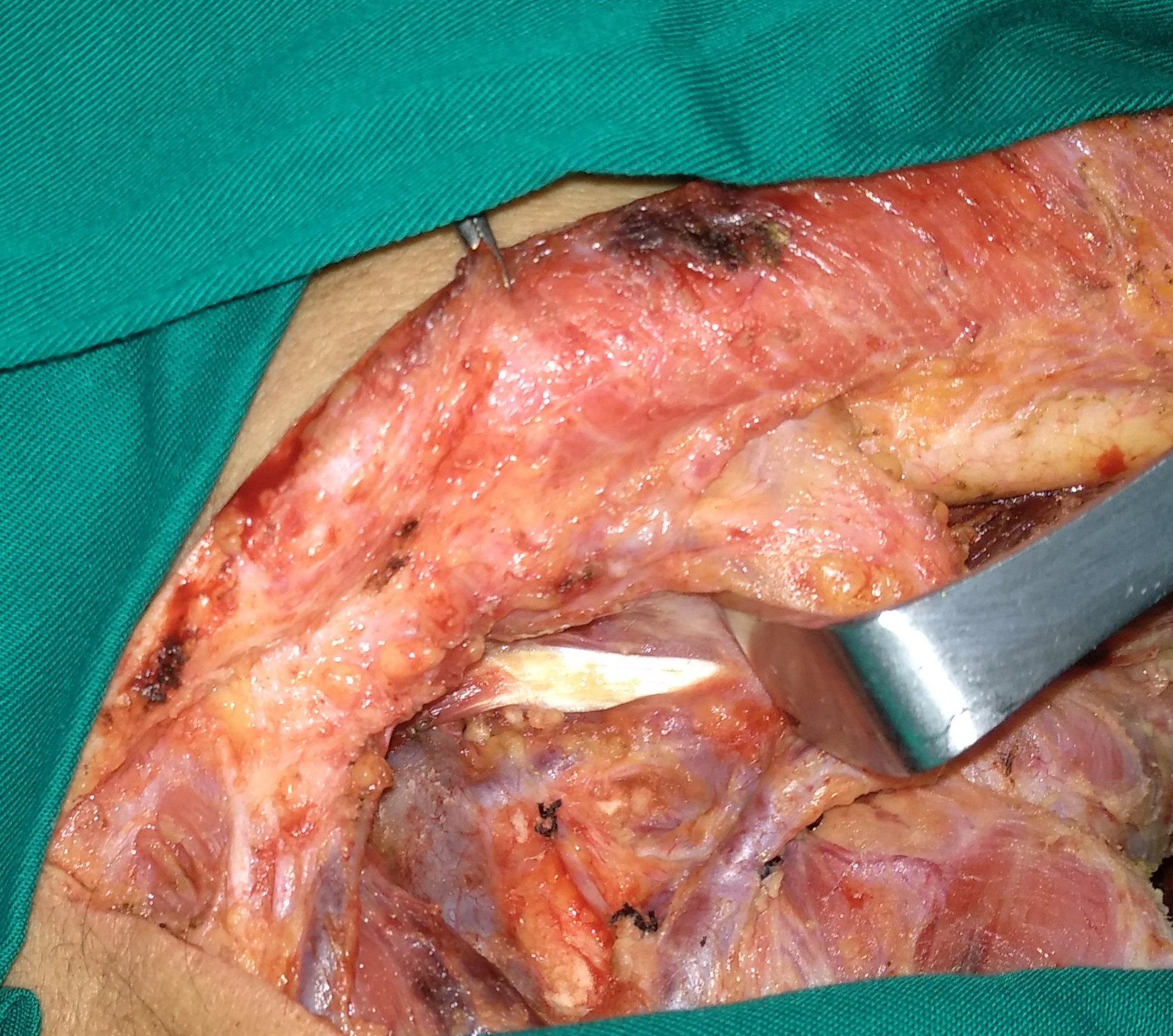

Supplement: Supplementary file 2 — Additional file 2 Supplemental Figure 2. The Posterior and superior parts were resected. [file 12885_2020_7534_MOESM2_ESM.jpg]
